# Rescue therapy within the UK Cystic Fibrosis Registry: An exploration of predictors of intravenous antibiotic use amongst adults with CF

**DOI:** 10.1111/resp.13174

**Published:** 2017-09-14

**Authors:** Zhe Hui Hoo, Martin J. Wildman, Rachael Curley, Stephen J. Walters, Michael J. Campbell

**Affiliations:** ^1^ School of Health and Related Research (ScHARR) University of Sheffield Sheffield UK; ^2^ Sheffield Adult CF Centre Northern General Hospital Sheffield UK

**Keywords:** cystic fibrosis, data interpretation, intravenous antibiotic, registry analysis, pulmonary exacerbation

## Abstract

**Background and objective:**

Intravenous (i.v.) antibiotics are needed for rescue when preventative therapy fails to achieve stability among adults with cystic fibrosis (CF). Understanding the distribution of i.v. days can provide insight into the care that adults with CF need. We aim to determine the baseline characteristics that are associated with higher i.v. use, in particular to test the hypothesis that prior‐year i.v. use is associated with future‐year i.v. use.

**Methods:**

This is a cross‐sectional analysis of the 2013–2014 UK CF registry data. Stepwise logistic regression was performed using current‐year i.v. days as the dependent variable, and demographic variables including prior‐year i.v. days as the covariates. Based on these results, study sample was divided into clinically meaningful subgroups using analysis similar to tree‐based method.

**Results:**

Data were available for 4269 adults in 2013 and 4644 adults in 2014. Prior‐year i.v. use was the strongest predictor for current‐year i.v. use followed by forced expiratory volume in 1 s (FEV_1_). Adults with high prior‐year i.v. use (>14 days) continued to require high levels of i.v., regardless of FEV_1_. Those with high prior‐year i.v. use and FEV_1_ ≥70% had higher current‐year i.v. days compared to adults with low prior‐year i.v. use and FEV_1_ <40% (28 days, interquartile range (IQR): 11–41 days vs 14 days, IQR: 0–28 days; Mann–Whitney P‐value <0.001 in 2013).

**Conclusion:**

CF people with prior high levels of rescue often continue to need high levels of rescue even if they have good FEV_1_. The reasons for this require further investigations.

## Abstract 2

Intravenous (i.v.) antibiotic is an important treatment option in cystic fibrosis and is also a marker of pulmonary exacerbations. Our study showed that previous‐year i.v. use is a strong predictor of current‐year i.v. use. This finding could help clinicians to identify people most at risk of future exacerbation.

**Abbreviations**
CF: cystic fibrosis
CFRD: CF‐related diabetes
CFTR: CF transmembrane regulator
FEV_1_: forced expiratory volume in 1 s
IQR: interquartile range
NHS: National Health Service
PERT: pancreatic replacement therapy
US CFFPR: US CF Foundation Patient Registry

## INTRODUCTION

Cystic fibrosis (CF) is an autosomal recessive genetic condition caused by mutations in the gene encoding the CF transmembrane regulator (CFTR) that affects around 10 000 people in the UK.^1^, ^2^ CF is a multisystem condition^1^ but lungs are the main organ affected, with CFTR dysfunction causing abnormal airway surface liquid.^1^, ^3^ People with CF are particularly susceptible to infection causing acute deterioration in lung health (i.e. pulmonary exacerbations), which leads to progressive lung damage and eventually respiratory failure.^4^

An important treatment option in CF is preventative inhaled therapies consisting of mucolytics and antibiotics, which have proven efficacy in reducing the frequency of exacerbations.^5^, ^6^ The effectiveness of these treatments is however limited by a real‐world medication adherence rates of 35–50%.^7^, ^8^ Acute treatment of pulmonary exacerbations has relatively limited research evidence.^9^ However, the importance of intravenous (i.v.) antibiotics in managing CF is indisputable—CF centres that use less antibiotics or have higher threshold for initiating i.v. in the face of an exacerbation were associated with people having lower lung function.^10^, ^11^ Indeed, i.v. antibiotics are recommended in all the major CF guidelines to treat exacerbations.^12^, ^13^, ^14^, ^15^

Understanding the clinical characteristics associated with i.v. antibiotics use is clinically important as it can provide insight into the care that people with CF need and the resources that are required for CF care. Exacerbation is also an important end point in CF clinical trials,^16^ and clinical factors associated with exacerbation could have implications for trial design.

A recent US CF Foundation Patient Registry (US CFFPR) data analysis has found that the frequency of prior‐year i.v.‐treated exacerbations is strongly associated with the frequency of future‐year i.v.‐treated exacerbations,^17^ even after adjustment for the other case‐mix factors, for example lung function.^17^, ^18^, ^19^ We analysed the 2013–2014 UK CF registry data to test whether the hypothesis that prior‐year i.v. use is associated with future‐year i.v. use, is also generalizable to the UK CF population.

## METHODS

This is a cross‐sectional analysis using the UK CF registry data for 2013–2014 from 28 UK adult CF centres. National Health Service (NHS) research ethics approval (Huntingdon Research Ethics Committee 07/Q0104/2) was granted for the UK CF Registry. Under the terms of the NHS ethics approval, the UK CF Trust steering committee approved this study.

People with lung transplantation and those on ivacaftor were excluded as both treatments have transformative effects on health outcomes,^20^, ^21^ such that their exacerbation rates and forced expiratory volume in 1 s (FEV_1_) no longer represent that of a typical adult with CF.

### Data

The following data were obtained

- Demographics: age, gender, CF centre identifier;
- Pancreatic status: people on pancreatic replacement therapy (PERT) were considered ‘pancreatic insufficient’ while those not on PERT were considered ‘pancreatic sufficient’;
- CF‐related diabetes: present (as defined by the UK CF Trust guideline),^22^ or not present;
- *Pseudomonas aeruginosa* status: no *P. aeruginosa* (negative cultures over a year), intermittent (positive cultures not fulfilling definition of chronic) or chronic (≥2 samples positive in one year);^23^
- Body mass index, BMI, in kg/m^2^;
- FEV_1_ during annual review (in % predicted, calculated with Knudson equation);^24^
- Annual total i.v. antibiotic days (in number of days).

Data were collected from people aged ≥16 years during annual reviews from January 2013 to December 2014. Since prior‐year i.v. use was a covariate in the analysis, i.v. use data for 2012 were also obtained.

Number of days on i.v. antibiotic, instead of number of i.v. courses, was chosen for analysis since it captures information on the cumulative i.v. antibiotic exposure to treat pulmonary exacerbations over a 1‐year period (not all i.v. courses are of the same duration).^25^

### Statistical analysis

Analyses were performed using SPSS v22 (IBM Corp, Armonk, NY, USA). Data for 2013 and 2014 were analysed separately to determine the consistency of any observations. The analysis consisted of two stages. First, logistic regression was performed to identify the order of the strength of association between the demographic and clinical variables with the current ‐ year i.v. use. Based on these results, the study sample was divided into clinically meaningful subgroups using analysis similar to tree‐based method^26^ for comparison of current‐year i.v. use between the subgroups.

Logistic regression was performed using current‐year i.v. days (≤14 days vs >14 days) as the dependent variable. 14‐Day was selected as the cut‐off since mean and median duration of each i.v. course in 2013 and 2014 was 14 days. The duration of a ‘standard’ i.v. course to treat an exacerbation is usually 14 days,^12^, ^15^ and people with ≥2 exacerbations per year (i.e. time between exacerbation <6 months^17^) have the most rapid FEV_1_ decline.^27^ Age, gender, CF centre (as a categorical variable), pancreatic status, CF‐related diabetes, intermittent *P. aeruginosa* (yes/no), chronic *P. aeruginosa* (yes/no), BMI, %FEV_1_ and prior‐year i.v. days (as a continuous variable) were the covariates. For this procedure, forward stepwise conditional analysis (probability for entry 0.05; probability for removal 0.10) was performed in a binary logistic regression model. The procedure began by identifying the covariate that was most strongly associated with current‐year i.v. use. The next strongest associated covariate was then selected after controlling for the first covariate. This continued until no further statistically significant covariates can be added to the model. Stepwise regression allows a relatively parsimonious model to be built even when several correlated covariates are present,^28^, ^29^ but it is essentially an exploratory analysis because it can lead to inflated Type I errors.^30^, ^31^

Therefore, the results from the stepwise logistic regression were subjected to a further analysis similar to tree‐based method to test the hypothesis that prior‐year i.v. use is associated with future‐year i.v. use. Tree‐based method is an efficient approach to understand the impact of risk factors for a condition where many potential confounders exist.^24^ For this analysis, the covariate most strongly associated with current‐year i.v. use as identified by stepwise logistic regression was used for the first ‘layer’ division of the study sample. Then, a further ‘layer’ of division for each generated subgroup was carried out using the next strongest associated covariate, and so on. For continuous covariates, clinical meaningful cut‐off points were used to divide the cohort instead of data‐driven optimal cut‐off points to avoid overfitting.^32^ For %FEV_1_, internationally accepted categories (<40%, 40–69.9% and ≥70%) were used as these categories are also applicable to the UK CF registry data.^33^ Prior‐year i.v. days were categorized into ≤14 days versus >14 days, similar to current‐year i.v. days. The current‐year i.v. days, as a continuous variable, were compared between subgroups at each ‘layer’ of the division using Mann–Whitney test or Jonckheere–Terpstra test depending on the number of subgroups, due to the non‐normal distribution of i.v. days. Jonckheere–Terpstra test is a generalization of the Mann–Whitney test for more than two ordered groups.

**Figure 1:**
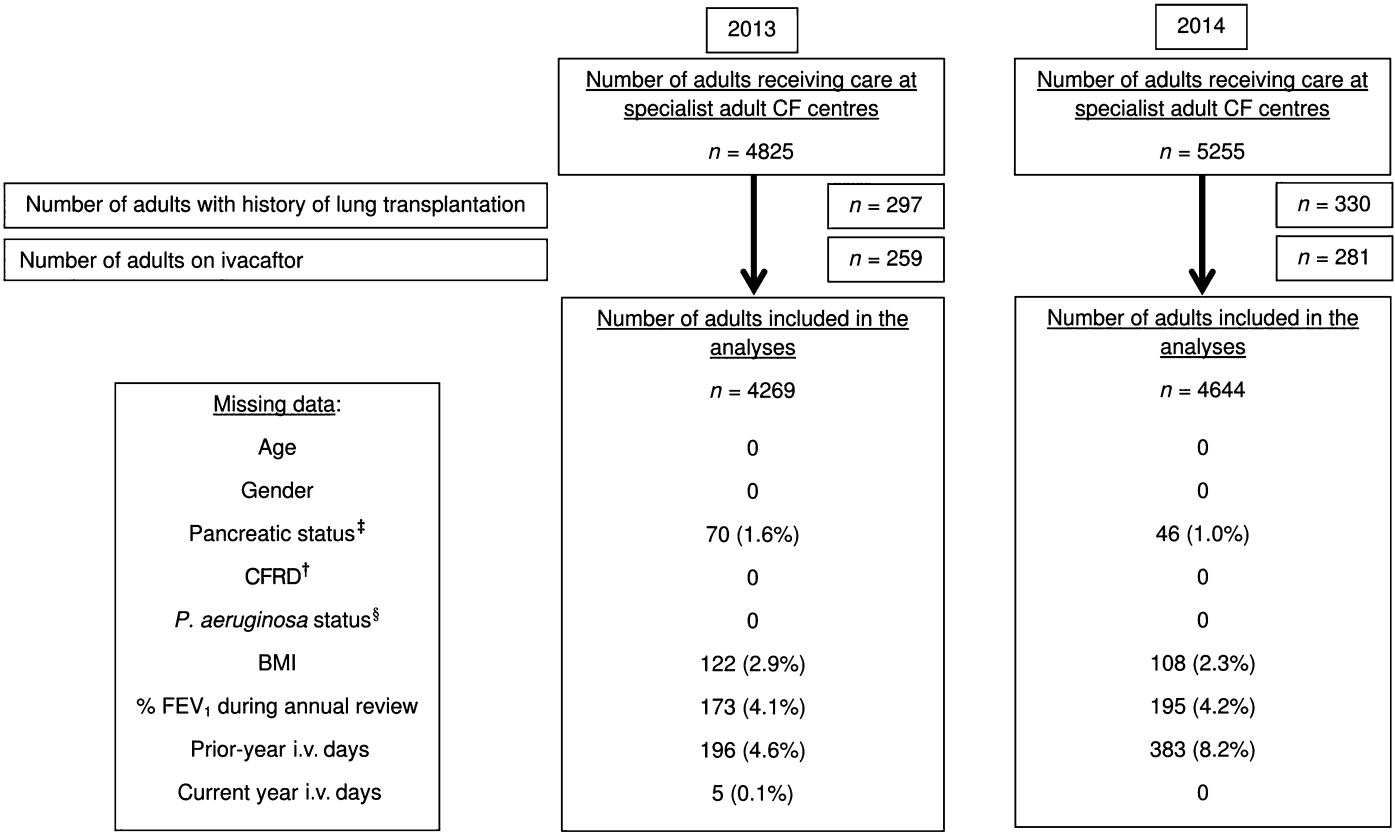
Summary of the number of adults included in the analyses and details regarding missing data. ^†^Cystic fibrosis (CF)‐related diabetes (CFRD) data are collected by the UK CF registry with only a check‐box for ‘CFRD present’. Therefore, if data are not available, it is assumed the person has no CFRD. It is therefore difficult to distinguish missing data from ‘no CFRD’ and hence missing data = 0. ^‡^Data for pancreatic replacement therapy (PERT) use were obtained. People on PERT were considered ‘pancreatic insufficient’. People not on PERT were considered ‘pancreatic sufficient’. PERT use documented as ‘unknown’ is considered as missing data. ^§^ P. aeruginosa status is collected by the UK CF registry with check‐boxes for ‘chronic’ and ‘intermittent’. Therefore, if data are not available, it is assumed the person has no P. aeruginosa. It is therefore difficult to distinguish missing data from ‘no P. aeruginosa’ and hence missing data = 0.

The analyses included those without any pulmonary exacerbations needing i.v. antibiotics (*n* = 1847, 43.3% of the sample for 2013; *n* = 2037, 43.9% of the sample for 2014) to obtain a more representative understanding of the adult CF population in the UK. *P*‐value <0.05 was considered to be statistically significant. Complete case approach was used as the extent of missing data (summarized in Fig. 1) was small. There were >1500 people with >14 days i.v. days for each year, which is more than adequate power for nine covariates in a binary logistic regression model.^34^ The study sample was also considerably larger than previous studies looking at factors associated with exacerbations^18^, ^19^ (except the recent US CFFPR analysis), which should allow adequate power for hypothesis testing between the different subgroups.

## RESULTS

A total of 4269 adults were included in the analysis for 2013 and 4644 for 2014, with 4874 study subjects in total across both years. In total, 103 453 i.v. days were used in 2013 and 111 981 i.v. days were used in 2014. Figure 1 summarizes the numbers of adults excluded and missing data. Baseline demographics stratified according to previous i.v. days was summarized in Table 1. Those with previous ‐ year i.v. use >14 days had lower FEV_1_ and higher current‐year i.v. use for both 2013 and 2014.

**Table 1:** Current year demographic and clinical characteristics stratified by prior‐year i.v. days

Demographics and clinical characteristics	2013			2014		
	Prior‐year i.v. days ≤ 14† *n* = 2433	Prior‐year i.v. days > 14† *n* = 1640	Overall *n* = 4269	Prior‐year i.v. days ≤ 14‡ *n* = 2586	Prior‐year i.v. days > 14‡ *n* = 1675	Overall *n* = 4644
Age (years) median (IQR)	28 (22–37)	27 (22–33)	28 (22–35)	28 (22–37)	27 (23–34)	28 (22–36)
Female (%)	995 (40.9)	849 (51.8)	1936 (45.4)	1066 (41.2)	877 (52.4)	2096 (45.1)
Pancreatic insufficient (%)	1858 (77.5)	1487 (92.0)	3449 (82.1)	1967 (77.0)	1526 (91.8)	3756 (81.7)
CF‐related diabetes (%)	568 (23.3)	760 (46.3)	1356 (31.8)	620 (24.0)	810 (48.4)	1525 (32.8)
*P. aeruginosa* status						
Chronic *P. aeruginosa* (%)	1068 (43.9)	1154 (70.4)	2276 (53.3)	1033 (39.9)	1167 (69.7)	2340 (50.4)
Intermittent *P. aeruginosa* (%)	350 (14.4)	201 (12.3)	576 (13.5)	422 (16.3)	183 (10.9)	689 (14.8)
BMI in kg/m^2^, mean (SD)	23.2 (3.9)	21.5 (3.6)	22.6 (3.9)	23.3 (3.9)	21.5 (3.5)	22.6 (3.9)
% predicted FEV_1_, mean (SD)	73.0 (23.4)	51.8 (21.1)	65.0 (24.8)	73.4 (23.6)	52.7 (21.4)	65.8 (25.0)
Current‐year i.v. days, median (IQR)	0 (0–14)	42 (16–67)	14 (0–35)	0 (0–14)	40 (15–66)	14 (0–34)

† As shown in Figure 1, there were 196 missing data for prior‐year i.v. days in 2013.

‡ As shown in Figure 1, there were 383 missing data for prior‐year i.v. days in 2014.

CF, cystic fibrosis; FEV_1_, forced expiratory volume in 1 s; IQR, interquartile range.

Graphs displaying the relationships between continuous covariates (age, BMI, %FEV_1_ and prior‐year i.v. days) with current‐year i.v. days are available in Appendix S1 (Supplementary Information). Contingency tables for all covariates are available in Appendix S2 (Supplementary Information).

For both 2013 and 2014, prior‐year i.v. use was the strongest predictor for current‐year i.v. use, followed by FEV_1_. Other covariates such as CF centre and pancreatic status were also associated with i.v. use, but the relationship was weaker and less consistent (Wald statistic <100) once prior‐year i.v. use and FEV_1_ had been taken into account (final model summarized in Table 2). Prior‐year i.v. use and FEV_1_ remained the strongest predictors for current‐year i.v. use in various sensitivity analyses (Appendix S3, Supplementary Information).

**Table 2:** Summary of the output from the final binary logistic regression model which include all nine covariates listed

2013 (3843 study subjects included in the analysis)				2014 (4040 study subjects included in the analysis)			
Covariates	Wald statistic	*P*‐value	Adjusted odds ratio (95% CI)	Covariates	Wald statistic	*P*‐value	Adjusted odds ratio (95% CI)
Prior‐year i.v. days	**449.5**	**<0.001**	1.06 (1.05–1.06)	Prior‐year i.v. days	**458.1**	**<0.001**	1.06 (1.05–1.06)
% Predicted FEV_1_	**140.1**	**<0.001**	0.97 (0.97–0.98)	% Predicted FEV_1_	**172.3**	**<0.001**	0.97 (0.97–0.98)
CF centre	90.3	<0.001		Pancreatic insufficient	32.5	<0.001	2.17 (1.66–2.83)
Pancreatic insufficient	9.6	<0.001	1.59 (1.19–2.13)	Female	32.4	<0.001	1.64 (1.38–1.95)
Chronic *P. aeruginosa*	21.9	<0.001	1.57 (1.30–1.89)	Chronic *P. aeruginosa*	30.9	<0.001	1.78 (1.45–2.18)
Female	15.9	<0.001	1.44 (1.20–1.71)	CF centre	67.6	<0.001	
Age (years)	17.6	<0.001	0.98 (0.97–0.99)	Intermittent *P. aeruginosa*	4.2	0.041	1.33 (1.01–1.75)
CF‐related diabetes	13.6	<0.001	1.44 (1.19–1.75)				

CF, cystic fibrosis; FEV_1_, forced expiratory volume in 1 s.

**Figure 2:**
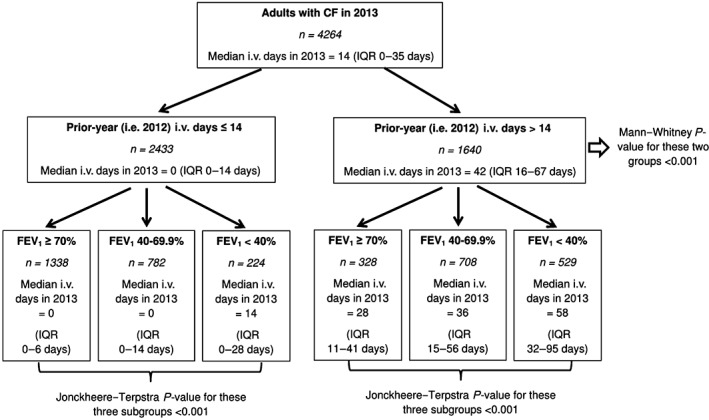
Tree‐based diagram for 2013 to summarize current‐year i.v. days according to the different clinical subgroups. CF, cystic fibrosis; FEV_1_, forced expiratory volume in 1 s; IQR, interquartile range.

**Figure 3:**
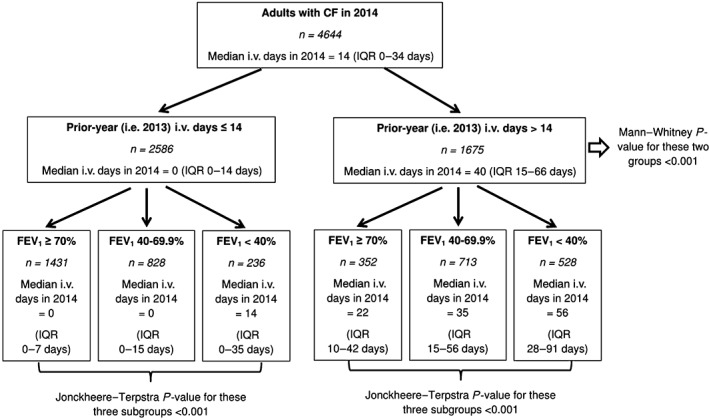
Tree‐based diagram for 2014 to summarize current‐year i.v. days according to the different clinical subgroups. CF, cystic fibrosis; FEV_1_, forced expiratory volume in 1 s; IQR, interquartile range.

Therefore, the cohort was divided into two groups based on prior‐year i.v. use (≤14 days and >14 days) and then each group is further divided into three subgroups based on %FEV_1_ (<40%, 40–69.9% and ≥70%) to generate six subgroups in total. The results of this analysis are summarized in Figures 2 and 3.

Current‐year i.v. use was clearly different between all the subgroups and the results were consistent for both 2013 and 2014. Adults with high prior‐year i.v. use (i.e. >14 days) continued to require high levels of i.v., even if they have good FEV_1_. Indeed, adults with high prior‐year i.v. use and FEV_1_ ≥ 70% had higher current‐year i.v. days compared to adults with low prior‐year i.v. use and FEV_1_ < 40% for both 2013 (28 days, interquartile range (IQR): 11–41 days vs 14 days, IQR: 0–28 days; *P* < 0.001) and 2014 (22 days, IQR: 10–42 days vs 14 days, IQR: 0–35 days; *P* = 0.003).

The clinical subgroups could be generated using different cut‐off points for prior‐year i.v. use but the results remained similar (see Appendix S4, Supplementary Information, for these sensitivity analyses).

## DISCUSSION

This study found that adults with high prior‐year i.v. use (>14 days) and low FEV_1_ (<40%) required the most i.v. among the six different clinical subgroups. In 2013, this group consisted 13.4% of the population but consumed 38.7% of i.v. days. Prior‐year i.v. use was a much stronger predictor of current‐year i.v. use compared to FEV_1_, such that those with high prior‐year i.v. use and high FEV_1_ (≥70%) still used more i.v. therapies compared to those with low prior‐year i.v. use (≤14 days) and low FEV_1_. The 2013 results were consistent with 2014 results, suggesting that chance is extremely unlikely to explain these results.

The finding that prior‐year i.v. use was the strongest independent predictor of current‐year i.v. use is consistent with the recent US CFFPR analysis.^17^ Whilst we acknowledge the limitations of retrospective observational registry‐based analyses that have been previously discussed,^17^, ^35^ the consistency and magnitude of the finding would suggest a genuine signal. The implications of this finding for clinical trials had also been discussed in detail—stratification of subject randomization by prior‐year i.v. use would be helpful to balance the baseline risk of pulmonary exacerbation. In fact, our randomized control trial to evaluate a complex self‐care intervention used this strategy for randomization.^36^

This finding also has other important clinical implications and could suggest possible differences in management strategies. Management of CF can be broadly dichotomized into ‘rescue’ therapy with i.v. antibiotics to treat pulmonary exacerbations and ‘prevention’ with inhaled therapies to minimize the risk of exacerbations.^37^ Whilst exacerbations may be stochastic events, the frequency and severity of exacerbations over a given period in a person with CF will be influenced by their clinical characteristics, environmental exposures and medical treatments.^38^ Given the efficacy of preventative inhaled therapies in reducing the frequency of exacerbations,^5^, ^6^ a possible explanation for high i.v. use would be the reliance on rescue therapies to compensate for insufficient utilization of preventative therapies.

Prior‐year i.v. use could also guide clinical strategy in managing CF. Insufficient utilization of preventative therapy could be due to low adherence, as median adherence with inhaled therapies in the real world is only 35–50%.^7^, ^8^, ^39^ Therefore, it is important to obtain objective adherence data wherever possible when assessing an adult with CF, especially if i.v. use is disproportionately high in relation to FEV_1_.

If objective nebulizer adherence is satisfactory, another cause for insufficient utilization of preventative therapy to consider is ‘therapeutic inertia’. Therapeutic inertia refers to the under‐prescription of efficacious treatments.^40^ In CF, the prescription of inhaled therapies have increased since mid 1990s.^41^, ^42^, ^43^ Adherence rates to international prescribing guidelines now exceed 90% for some specialist CF centres.^44^ If i.v. requirement is still disproportionately high but objective adherence is high and no obvious complications are found, escalation of preventative therapies should be considered. An example would be the initiation of inhaled antibiotic treatment for someone who is only on inhaled mucolytics. The detection of *P. aeruginosa* infection would typically prompt the prescription of inhaled antibiotics. However, there may be merit in using long‐term inhaled antibiotics, even when *P. aeruginosa* is not detected, if there is a history of frequent exacerbations.^45^ This is a strategy that has some evidence of success in non‐CF bronchiectasis.^46^

Our finding highlights potential limitations of FEV_1_ as a marker of lung health. Low FEV_1_ is a known poor prognostic marker in CF and is strongly associated with mortality.^47^, ^48^ FEV_1_ trend is therefore a commonly used primary end point in CF clinical trials and an important parameter measured during annual reviews as a marker of lung health for adults with CF.^16^, ^49^ However, as the health of people with CF improved over time, the sensitivity of FEV_1_ as a marker of lung health decreases.^50^ This study found a group of people with high i.v. use year on year, yet who maintained relatively good lung function (FEV_1_ > 70%). It may be possible to initially fend off FEV_1_ decline despite frequent exacerbations by simply relying on prompt and aggressive rescue i.v. treatments. Such strategy is likely to result in wild fluctuations of FEV_1_, with high FEV_1_ post i.v. treatment not sustained due to lack of preventative treatments. A trend of declining FEV_1_ tends to be predated by increased FEV_1_ variability^51^; hence, FEV_1_ variability is a more sensitive marker of lung health. There is increasing evidence regarding the prognostic impact of FEV_1_ variability,^51^, ^52^ but we were not able to study the correlation between FEV_1_ variability and i.v. use as the UK CF registry does not routinely collect encounter‐based FEV_1_ data.

In conclusion, exacerbations as indicated by i.v. use are clinically important events in CF with long‐term impact on morbidity and mortality. Prevention of exacerbations is a priority due to the risk of FEV_1_ decline and lung damage even with intensive i.v. antibiotics, hence the importance of understanding the predictors of frequent exacerbations. This UK CF registry analysis used i.v. days as a marker of the frequency and severity of exacerbations, and found that current‐year i.v. use is most strongly associated with prior‐year i.v. use. A recent US CFFPR data analysis also found similar results.^17^ That means those with history of frequent exacerbations continue to remain most at risk for future exacerbations, even with good FEV_1_. Inadequate utilization of preventative inhaled therapies may be one of the reasons for this reliance on rescue therapies. Therefore, high i.v. use should prompt clinicians to assess both the adherence rate and the prescribed inhaled therapies regime.

## Disclosure statement

Part of the analysis in this paper has been presented as an oral presentation in the 2016 European Cystic Fibrosis Society Conference. The abstract is published in the *Journal of Cystic Fibrosis* 2016, Vol 15, Suppl 1, entitled Rescue therapy within the UK CF registry: an exploration of the predictors of i.v. antibiotic use amongst adults with CF.

## Supporting information


**Appendix S1** The relationships between age, BMI, % forced expiratory volume in 1 s and prior‐year i.v. days with current‐year i.v. days for 2013 and 2014.
**Appendix S2** A contingency table showing the distribution of covariates according to current‐year i.v. days.
**Appendix S3** Sensitivity analyses using number of i.v. courses (instead of i.v. days) as the dependent variable in a logistic regression model and using ordinal regression models to explore different cut‐off points for i.v. courses and i.v. days.
**Appendix S4** Sensitivity analyses using number of i.v. courses (instead of i.v. days) and different cut‐off points to generate the clinical subgroups.

## References

[1] Elborn JS . Cystic fibrosis. Lancet 2016; 388: 2519–31. 27140670 10.1016/S0140-6736(16)00576-6

[2] The UK CF Registry Steering Committee . UK Cystic Fibrosis Registry 2015 Annual Data Report, 2016 [Accessed 31 Jan 2017.] Available from URL:https://www.cysticfibrosis.org.uk/the-work-we-do/uk-cf-registry/reporting-and-resources

[3] Haq IJ , Gray MA , Garnett JP , Ward C , Brodlie M . Airway surface liquid homeostasis in cystic fibrosis: pathophysiology and therapeutic targets. Thorax 2016; 71: 284–7. 26719229 10.1136/thoraxjnl-2015-207588

[4] Goss CH , Burns JL . Exacerbations in cystic fibrosis. 1: epidemiology and pathogenesis. Thorax 2007; 62: 360–7. 17387214 10.1136/thx.2006.060889 PMC2092469

[5] Ryan G , Singh M , Dwan K . Inhaled antibiotics for long‐term therapy in cystic fibrosis. Cochrane Database Syst. Rev. 2011; 3: CD001021. 10.1002/14651858.CD001021.pub2 21412868

[6] Yang C , Chilvers M , Montgomery M , Nolan SJ . Dornase alfa for cystic fibrosis. Cochrane Database Syst. Rev. 2016; 4: CD001127. 27043279 10.1002/14651858.CD001127.pub3

[7] Daniels T , Goodacre L , Sutton C , Pollard K , Conway S , Peckham D . Accurate assessment of adherence: self‐report and clinician report vs electronic monitoring of nebulizers. Chest 2011; 140: 425–32. 21330381 10.1378/chest.09-3074

[8] Quittner AL , Zhang J , Marynchenko M , Chopra PA , Signorovitch J , Yushkina Y , Riekert KA . Pulmonary medication adherence and health‐care use in cystic fibrosis. Chest 2014; 146: 142–51. 24480974 10.1378/chest.13-1926

[9] Hurley MN , Prayle AP , Flume P . Intravenous antibiotics for pulmonary exacerbations in people with cystic fibrosis. Cochrane Database Syst. Rev. 2015; 7: CD009730. 10.1002/14651858.CD009730.pub2 PMC6481905 26226131

[10] Johnson C , Butler SM , Konstan MW , Morgan W , Wohl ME . Factors influencing outcomes in cystic fibrosis: a center‐based analysis. Chest 2003; 123: 20–7. 12527598 10.1378/chest.123.1.20

[11] Schechter MS , Regelmann WE , Sawicki GS , Rasouliyan L , VanDevanter DR , Rosenfeld M , Pasta D , Morgan W , Konstan MW . Antibiotic treatment of signs and symptoms of pulmonary exacerbations: a comparison by care site. Pediatr. Pulmonol. 2015; 50: 431–40. 25530325 10.1002/ppul.23147

[12] UK Cystic Fibrosis Trust Antibiotic Working Group . Antibiotic treatment for cystic fibrosis. 2009 [Accessed 31 Jan 2017.] Available from URL: https://www.cysticfibrosis.org.uk/the-work-we-do/clinical-care/consensus-documents

[13] Doring G , Flume P , Heijerman H , Elborn JS ; Consensus Study Group . Treatment of lung infection in patients with cystic fibrosis: current and future strategies. J. Cyst. Fibros. 2012; 11: 461–79. 23137712 10.1016/j.jcf.2012.10.004

[14] Flume PA , Mogayzel PJ Jr , Robinson KA , Goss CH , Rosenblatt RL , Kuhn RJ , Marshall BC ; Clinical Practice Guidelines for Pulmonary Therapies Committee . Cystic fibrosis pulmonary guidelines: treatment of pulmonary exacerbations. Am. J. Respir. Crit. Care Med. 2009; 180: 802–8. 19729669 10.1164/rccm.200812-1845PP

[15] Kerem E , Conway S , Elborn S , Heijerman H ; Consensus Committee . Standards of care for patients with cystic fibrosis: a European consensus. J. Cyst. Fibros. 2005; 4: 7–26. 10.1016/j.jcf.2004.12.002 15752677

[16] VanDevanter DR , Konstan MW . Outcome measures for clinical trials assessing treatment of cystic fibrosis lung disease. Clin. Investig. (Lond.) 2012; 2: 163–75. 10.4155/cli.11.174 PMC4486293 26146539

[17] VanDevanter DR , Morris NJ , Konstan MW . i.v.‐treated pulmonary exacerbations in the prior year: an important independent risk factor for future pulmonary exacerbation in cystic fibrosis. J. Cyst. Fibros. 2016; 15: 372–9. 26603642 10.1016/j.jcf.2015.10.006 PMC4841746

[18] Block JK , Vandemheen KL , Tullis E , Fergusson D , Doucette S , Haase D , Berthiaume Y , Brown N , Wilcox P , Bye P *et al.* Predictors of pulmonary exacerbations in patients with cystic fibrosis infected with multi‐resistant bacteria. Thorax 2006; 61: 969–74. 16844728 10.1136/thx.2006.061366 PMC2121166

[19] Jarad NA , Giles K . Risk factors for increased need for intravenous antibiotics for pulmonary exacerbations in adult patients with cystic fibrosis. Chron. Respir. Dis. 2008; 5: 29–33. 18303099 10.1177/1479972307085635

[20] Pego‐Fernandes PM , Abrao FC , Fernandes FL , Caramori ML , Samano MN , Jatene FB . Spirometric assessment of lung transplant patients: one year follow‐up. Clinics (Sao Paulo) 2009; 64: 519–25. 19578655 10.1590/S1807-59322009000600006 PMC2705150

[21] Ramsey BW , Davies J , McElvaney NG , Tullis E , Bell SC , Dřevínek P , Griese M , McKone EF , Wainwright CE , Konstan MW *et al*; VX08‐770‐102 Study Group . A CFTR potentiator in patients with cystic fibrosis and the G551D mutation. *N. Engl. J. Med.* 2011; **365:** 1663–72. 10.1056/NEJMoa1105185 PMC3230303 22047557

[22] The UK Cystic Fibrosis Trust Diabetes Working Group . Management of cystic fibrosis related diabetes mellitus, 2004 [Accessed 30 May 2017.] Available from URL: https://www.cysticfibrosis.org.uk/the-work-we-do/clinical-care/consensus-documents

[23] Goss CH , MacNeill SJ , Quinton HB , Marshall BC , Elbert A , Knapp EA , Petren K , Gunn E , Osmond J , Bilton D . Children and young adults with CF in the USA have better lung function compared with the UK. Thorax 2015; 70: 229–36. 25256255 10.1136/thoraxjnl-2014-205718 PMC4838510

[24] Knudson RJ , Lebowitz MD , Holberg CJ , Burrows B . Changes in the normal maximal expiratory flow‐volume curve with growth and aging. Am. Rev. Respir. Dis. 1983; 127: 725–34. 6859656 10.1164/arrd.1983.127.6.725

[25] Sequeiros IM , Jarad NA . Extending the course of intravenous antibiotics in adult patients with cystic fibrosis with acute pulmonary exacerbations. Chron. Respir. Dis. 2012; 9: 213–20. 22637747 10.1177/1479972312445903

[26] Zhang H , Bracken MB . Tree‐based, two‐stage risk factor analysis for spontaneous abortion. Am. J. Epidemiol. 1996; 144: 989–96. 8916510 10.1093/oxfordjournals.aje.a008869

[27] Waters V , Stanojevic S , Atenafu EG , Lu A , Yau Y , Tullis E , Ratjen F . Effect of pulmonary exacerbations on long‐term lung function decline in cystic fibrosis. Eur. Respir. J. 2012; 40: 61–6. 22135280 10.1183/09031936.00159111

[28] Uh HW , Mertens BJ , Jan van der Wijk H , Putter H , van Houwelingen HC , Houwing‐Duistermaat JJ . Model selection based on logistic regression in a highly correlated candidate gene region. BMC Proc. 2007; 1: S114. 18466455 10.1186/1753-6561-1-s1-s114 PMC2367469

[29] Zhang Z . Variable selection with stepwise and best subset approaches. Ann. Transl. Med. 2016; 4: 136. 27162786 10.21037/atm.2016.03.35 PMC4842399

[30] Braun MT , Oswald FL . Exploratory regression analysis: a tool for selecting models and determining predictor importance. Behav. Res. Methods 2011; 43: 331–9. 21298571 10.3758/s13428-010-0046-8

[31] Mundry R , Nunn CL . Stepwise model fitting and statistical inference: turning noise into signal pollution. Am. Nat. 2009; 173: 119–23. 19049440 10.1086/593303

[32] Leeflang MM , Moons KG , Reitsma JB , Zwinderman AH . Bias in sensitivity and specificity caused by data‐driven selection of optimal cutoff values: mechanisms, magnitude, and solutions. Clin. Chem. 2008; 54: 729–37. 18258670 10.1373/clinchem.2007.096032

[33] Hoo ZH , Daniels T , Wildman MJ , Teare MD , Bradley JM . Airway clearance techniques used by people with cystic fibrosis in the UK. Physiotherapy 2015; 101: 340–8. 25910514 10.1016/j.physio.2015.01.008

[34] Peduzzi P , Concato J , Kemper E , Holford TR , Feinstein AR . A simulation study of the number of events per variable in logistic regression analysis. J. Clin. Epidemiol. 1996; 49: 1373–9. 8970487 10.1016/s0895-4356(96)00236-3

[35] Urschel S . Apples, oranges, and statistical magic: limitations of registry studies and need for collaborative studies. J. Heart Lung Transplant. 2015; 34: 1136–8. 26143665 10.1016/j.healun.2015.05.013

[36] Wildman MJ . Development and evaluation of an intervention to support Adherence to treatment in adults with Cystic Fibrosis (ACtiF), 2016 [Accessed 31 Jan 2017.] Available from URL: https://www.sheffield.ac.uk/scharr/sections/hsr/mcru/actif.

[37] Wildman MJ , Hoo ZH . Moving cystic fibrosis care from rescue to prevention by embedding adherence measurement in routine care. Paediatr. Respir. Rev. 2014; 15(Suppl. 1): 16–8. 24835307 10.1016/j.prrv.2014.04.007

[38] Wolfenden LL , Schechter MS . Genetic and non‐genetic determinants of outcomes in cystic fibrosis. Paediatr. Respir. Rev. 2009; 10: 32–6. 19203742 10.1016/j.prrv.2008.04.002

[39] Hoo ZH , Curley R , Walters SJ , Campbell MJ , Wildman MJ . Improving nebuliser adherence in an adult CF centre – differences in unadjusted adherence vs normative adherence [abstract]. Pediatr. Pulmonol. 2016; 51: S449. [Abstract ID – 667].

[40] Allen JD , Curtiss FR , Fairman KA . Nonadherence, clinical inertia, or therapeutic inertia? J. Manag. Care Pharm. 2009; 15: 690–5. 19803559 10.18553/jmcp.2009.15.8.690 PMC10437577

[41] Dasenbrook EC , Konstan MW , VanDevanter DR . Association between the introduction of a new cystic fibrosis inhaled antibiotic class and change in prevalence of patients receiving multiple inhaled antibiotic classes. J. Cyst. Fibros. 2015; 14: 370–5. 25496726 10.1016/j.jcf.2014.11.005 PMC4417393

[42] Konstan MW , VanDevanter DR , Rasouliyan L , Pasta DJ , Yegin A , Morgan WJ , Wagener JS ; Scientific Advisory Group ; Investigators and Coordinators of the Epidemiologic Study of Cystic Fibrosis . Trends in the use of routine therapies in cystic fibrosis: 1995‐2005. Pediatr. Pulmonol. 2010; 45: 1167–72. 20717935 10.1002/ppul.21315 PMC4112572

[43] Quinton HB , O'Connor GT . Current issues in quality improvement in cystic fibrosis. Clin. Chest Med. 2007; 28: 459–72. 17467560 10.1016/j.ccm.2007.02.013

[44] Moore BM , Laguna TA , Liu M , McNamara JJ . Increased adherence to CFF practice guidelines for pulmonary medications correlates with improved FEV1. Pediatr. Pulmonol. 2013; 48: 747–53. 22997186 10.1002/ppul.22665 PMC3856882

[45] Hoo ZH , Curley R , Campbell MJ , Walters SJ , Hind D , Wildman MJ . Accurate reporting of adherence to inhaled therapies in adults with cystic fibrosis: methods to calculate “normative adherence”. Patient. Prefer. Adherence 2016; 10: 887–900. 27284242 10.2147/PPA.S105530 PMC4883819

[46] Nadig TR , Flume PA . Aerosolized antibiotics for patients with bronchiectasis. Am. J. Respir. Crit. Care Med. 2016; 193: 808–10. 27035784 10.1164/rccm.201507-1449LE

[47] Kerem E , Reisman J , Corey M , Canny GJ , Levison H . Prediction of mortality in patients with cystic fibrosis. N. Engl. J. Med. 1992; 326: 1187–91. 1285737 10.1056/NEJM199204303261804

[48] Liou TG , Adler FR , Fitzsimmons SC , Cahill BC , Hibbs JR , Marshall BC . Predictive 5‐year survivorship model of cystic fibrosis. Am. J. Epidemiol. 2001; 153: 345–52. 11207152 10.1093/aje/153.4.345 PMC2198936

[49] Long JM , Fauset‐Jones J , Dixon MJ , Worthington‐Riley D , Sharma V , Patel L , David TJ . Annual review hospital visits for patients with cystic fibrosis. J. R. Soc. Med. 2001; 94(Suppl. 40): 12–6. 11601158 10.1177/014107680109440s05 PMC1310580

[50] Stanojevic S , Ratjen F . Physiologic endpoints for clinical studies for cystic fibrosis. J. Cyst. Fibros. 2016; 15: 416–23. 27316663 10.1016/j.jcf.2016.05.014

[51] Morgan WJ , VanDevanter DR , Pasta DJ , Foreman AJ , Wagener JS , Konstan MW ; Scientific Advisory Group ; Investigators and Coordinators of Epidemiologic Study of Cystic Fibrosis . Forced expiratory volume in 1 second variability helps identify patients with cystic fibrosis at risk of greater loss of lung function. J. Pediatr. 2016; 169: 116–21. 26388208 10.1016/j.jpeds.2015.08.042

[52] Adler FR , Liou TG . The dynamics of disease progression in cystic fibrosis. PLoS One 2016; 11: e0156752. 27248696 10.1371/journal.pone.0156752 PMC4889102

