# Supplementary material for: Rescue therapy within the UK Cystic Fibrosis Registry: An exploration of predictors of intravenous antibiotic use amongst adults with CF
Source: Respirology. 2017 Sep 14;23(2):190–7. doi: 10.1111/resp.13174 (PMC5813205; doi:10.1111/resp.13174)
Supplement: Supplementary file 1 — Appendix S1 The relationships between age, BMI, % forced expiratory volume in 1 s and prior‐year i.v. days with current‐year i.v. days for 2013 and 2014. Appendix S2 A contingency table showing the distribution of covariates according to current‐year i.v. days. Appendix S3 Sensitivity analyses using number of i.v. courses (instead of i.v. days) as the dependent variable in a logistic regression model and using ordinal regression models to explore different cut‐off points for i.v. courses and i.v. days. Appendix S4 Sensitivity analyses using number of i.v. courses (instead of i.v. days) and different cut‐off points to generate the clinical subgroups. [file RESP-23-190-s001.docx]

**SUPPLEMENTARY INFORMATION**

**Rescue therapy within the UK Cystic Fibrosis Registry: An exploration of predictors of intravenous antibiotic use amongst adults with CF**

Zhe Hui Hoo^1,2^, Martin James Wildman^2,1^, Rachael Curley^2,1^, Stephen John Walters^1^, Michael Joseph Campbell^1^

^1^ School of Health and Related Research (ScHARR), University of Sheffield, Sheffield, UK

^2^ Sheffield Adult CF Centre, Northern General Hospital, Sheffield, UK

**Appendix S1** *The relationships between age, BMI, % FEV1 & prior-year IV days with current year IV days for 2013 and 2014*

**Appendix S2** *A contingency table showing the distribution of covariates according to current year IV days*

**Appendix S3** *Sensitivity analyses using number of IV courses (instead of IV days) as the dependent variable in a logistic regression model and using ordinal regression models to explore different cut-offs for IV courses & IV days*

**Table S3.1** *Summary of the output from the final binary logistic regression model which include all nine covariates listed*

**Table S3.2** *Summary of the output from the ordinal logistic regression model which include all nine covariates listed*

**Table S3.3** *Summary of the output from the ordinal logistic regression model which include all nine covariates listed*

**Appendix S4** *Sensitivity analyses using number of IV courses (instead of IV days) and different cut-offs to generate the clinical subgroups*

**Figure S4.1** *Tree-based diagram for 2013 to summarise current year IV days according to the different clinical subgroups using two categories of prior-year IV courses (≤ 1 course vs ≥ 2 courses)*

**Figure S4.2** *Tree-based diagram for 2013 to summarise current year IV days according to the different clinical subgroups using four categories of prior-year IV courses (no IV, 1 course, 2-3 courses, ≥ 4 courses)*

**Figure S4.3** *Tree-based diagram for 2013 to summarise current year IV days according to the different clinical subgroups using four categories of prior-year IV days (no IV, 1-17 days, 18-42 days, ≥ 43 days)*

**Figure S4.4** *Tree-based diagram for 2014 to summarise current year IV days according to the different clinical subgroups using two categories of prior-year IV courses (≤ 1 course vs ≥ 2 courses)*

**Figure S4.5** *Tree-based diagram for 2014 to summarise current year IV days according to the different clinical subgroups using four categories of prior-year IV courses (no IV, 1 course, 2-3 courses, ≥ 4 courses)*

**Figure S4.6** *Tree-based diagram for 2014 to summarise current year IV days according to the different clinical subgroups using four categories of prior-year IV days (no IV, 1-17 days, 18-42 days, ≥ 43 days)*

**Appendix S1: The relationships between age, BMI, % FEV_1_ & prior-year IV days with current year IV days for 2013 and 2014**


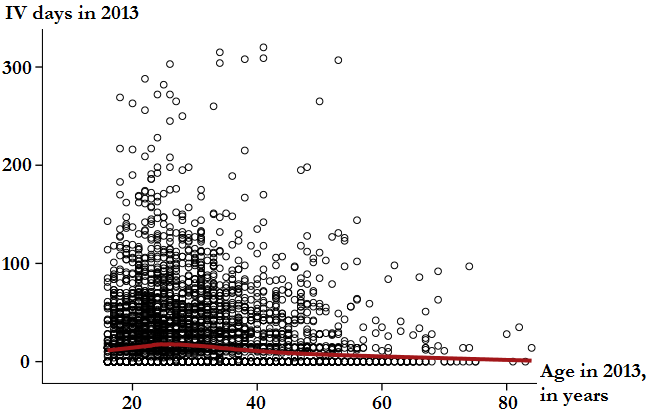

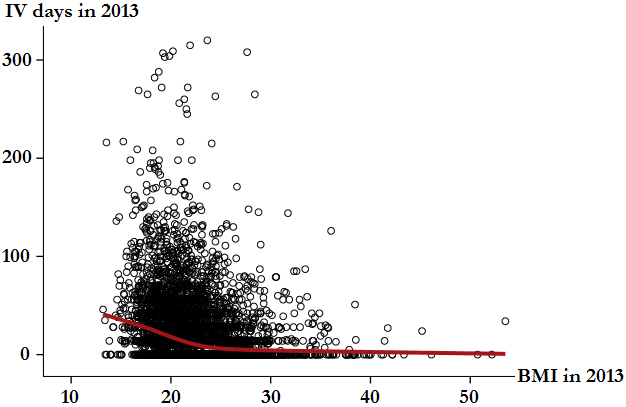
 Scatter plot of IV days in 2013 vs age Scatter plot of IV days in 2013 vs BMI

Current year IV days showed no clear relationship with age Current year IV days was negatively correlated with BMI


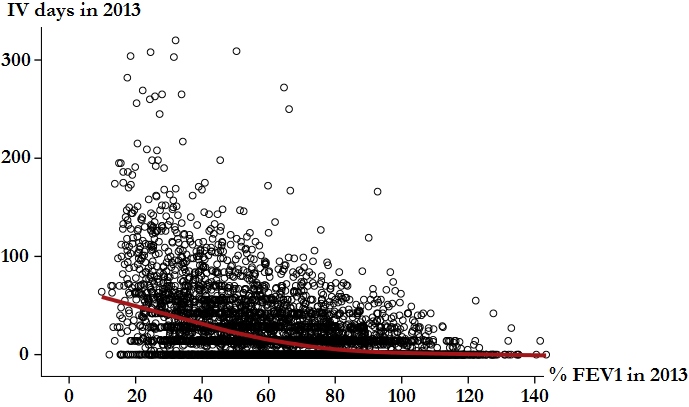
 Scatter plot of IV days in 2013 vs % FEV_1_ Scatter plot of IV days in 2013 vs IV days in 2012


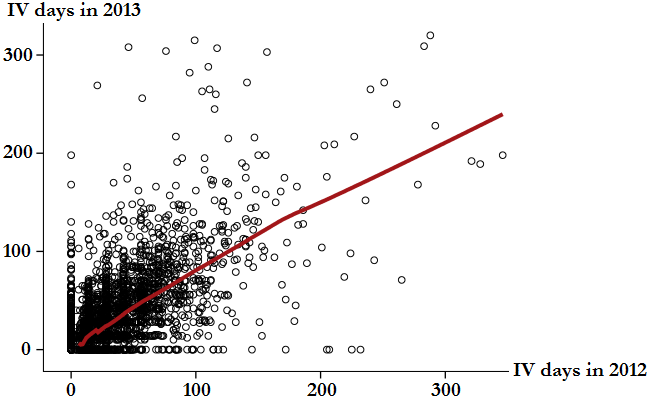


Current year IV days was negatively correlated with % FEV_1_ Current year IV days was positively correlated with prior-year IV days

Scatter plot of IV days in 2014 vs age Scatter plot of IV days in 2014 vs BMI


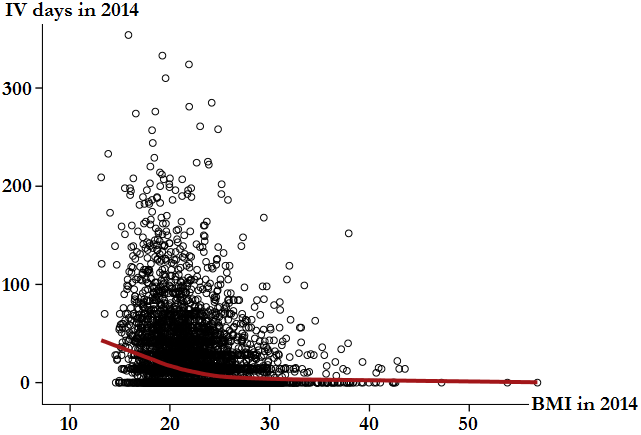

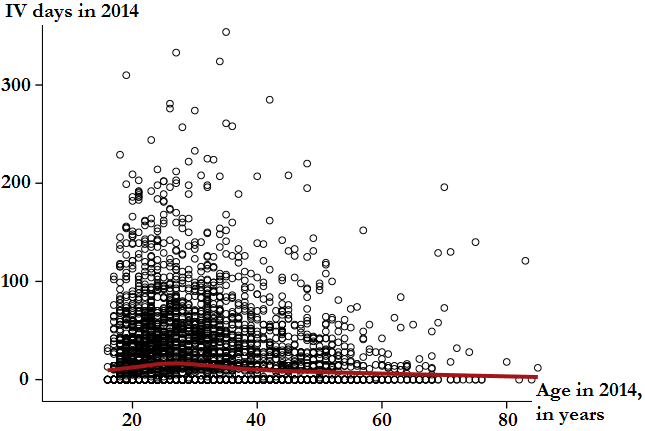


Current year IV days showed no clear relationship with age Current year IV days was negatively correlated with BMI


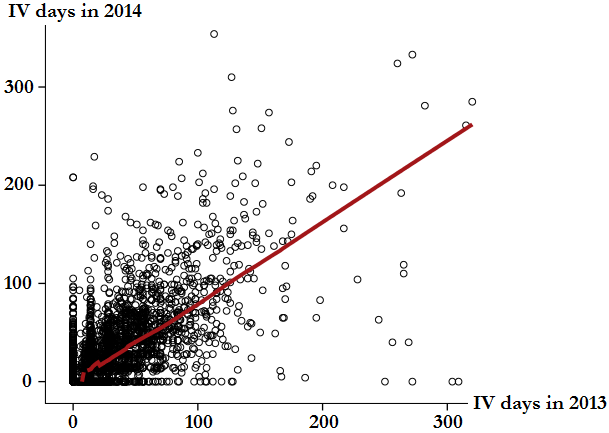

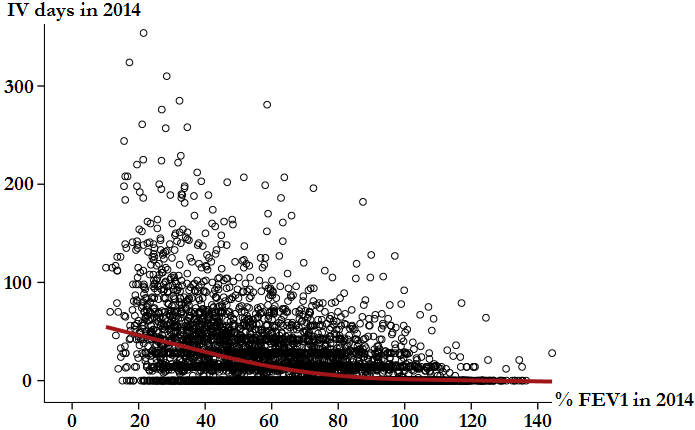
 Scatter plot of IV days in 2014 vs % FEV_1_ Scatter plot of IV days in 2014 vs IV days in 2013

Current year IV days was negatively correlated with % FEV_1_ Current year IV days was positively correlated with prior-year IV days

*The Local Polynomial Regression (LOESS) curve is a non-parametric method for fitting smooth curves to empirical data, to depict relationships between variables. For reference, see:

Cleveland WS, Devlin SJ. Locally weighted regression: an approach to regression analysis by local fitting. *J Am Stat Assoc* 1988; 83: 596–610.

**Appendix S2: A contingency table showing the distribution of covariates according to current year IV days**

| Demographics and clinical characteristics (covariates): | 2013 | | 2014 | |
| --- | --- | --- | --- | --- |
|  | Current year IV days ≤ 14  N = 2542 | Current year IV days > 14  N = 1722 | Current year IV days ≤ 14  N = 2772 | Current year IV days > 14  N = 1872 |
| Age in years, median (IQR)  Female, %  Pancreatic insufficient, %  CF related diabetes, %  *P. aeruginosa* status  Chronic *P. aeruginosa*, %  Intermittent *P. aeruginosa*, %  BMI in kg/m^2^, median (IQR)  % predicted FEV1, median (IQR)  Prior-year IV days, median (IQR) | 28 (22 – 37)  1043 (41.0%)  1889 (75.7%)  562 (22.1%)  1065 (41.9%)  364 (14.3%)  22.8 (20.7 – 25.3)  75.0 (56.7 – 90.6)  0 (0 – 14) | 27 (22 – 33)  891 (51.7%)  1558 (91.9%)  793 (46.1%)  1211 (70.3%)  212 (12.3%)  21.1 (19.1 – 23.3)  49.1 (34.7 – 66.8)  37 (15 – 60) | 28 (22 – 37)  1123 (40.5%)  2045 (74.7%)  654 (23.6%)  1049 (37.8%)  455 (16.4%)  22.8 (20.7 – 25.3)  76.3 (58.3 – 91.8)  0 (0 – 14) | 28 (23 – 34)  973 (52.0%)  1711 (91.9%)  871 (46.5%)  1291 (69.0%)  234 (12.5%)  21.2 (19.1 – 23.4)  49.7 (35.1 – 66.8)  35 (14 – 59) |

**Appendix S3: Sensitivity analyses using number of IV courses (instead of IV days) as the dependent variable in a logistic regression model and using ordinal regression models to explore different cut-offs for IV courses & IV days**

*Sensitivity analysis 1: using number of IV courses in a stepwise logistic regression model*

Using number of IV courses in the current year (≤1 course vs ≥2 courses) as the dependent variable in a stepwise binary logistic regression model as described in the ‘Methods’, prior-year IV use was the strongest predictor for current year IV use followed by FEV_1_ for both 2013 and 2014. The final model is summarised in Table S3.1. These results show that prior-year IV use and FEV_1_ were the most robust predictors for current year IV use. The results were not influenced by considering cumulative IV exposure (as IV days) or the number of IV courses as the outcome.

**Table S3.1**: Summary of the output from the final binary logistic regression model which include all nine covariates listed

| **2013** (3843 study subjects included in the analysis) | | | | **2014** (4040 study subjects included in the analysis) | | | |
| --- | --- | --- | --- | --- | --- | --- | --- |
| Covariates: | Wald statistic | P-value | Adjusted odds ratio  (95% CI) | Covariates: | Wald statistic | P-value | Adjusted odds ratio  (95% CI) |
| **Prior-year IV days**  **% predicted FEV1**  CF centre  Age in years  Chronic *P. aeruginosa*  Female  CF related diabetes  Pancreatic insufficient | **437.6**  **136.4**  107.4  20.2  23.4  21.8  10.1  8.4 | **< 0.001**  **< 0.001**  < 0.001  < 0.001  < 0.001  < 0.001  0.001  0.004 | 1.05 (1.05 – 1.06)  0.97 (0.97 – 0.98)  0.98 (0.97 – 0.99)  1.61 (1.33 – 1.95)  1.54 (1.28 – 1.84)  1.37 (1.13 – 1.67)  1.57 (1.16 – 2.13) | **Prior-year IV days**  **% predicted FEV1**  Chronic *P. aeruginosa*  CF centre  Female  Pancreatic insufficient  BMI in kg/m^2^  Intermittent *P. aeruginosa*  CF related diabetes | **441.3**  **132.7**  34.9  89.6  28.9  12.7  10.4  5.2  4.4 | **< 0.001**  **< 0.001**  < 0.001  < 0.001  < 0.001  < 0.001  0.001  0.023  0.035 | 1.05 (1.05 – 1.06)  0.98 (0.97 – 0.98)  1.90 (1.54 – 2.35)  1.62 (1.36 – 1.93)  1.69 (1.27 – 2.26)  0.96 (0.93 – 0.98)  1.39 (1.05 – 1.85)  1.23 (1.01 – 1.48) |

*Sensitivity analysis 2: using four categories of IV courses in an ordinal regression model*

Using number of IV courses in the current year (no IV, 1 course, 2-3 courses, ≥4 courses) as the dependent variable in an ordinal logistic regression model with the same co-variates as described in the ‘Methods’, prior-year IV use remained the strongest predictor for current year IV use followed by FEV_1_ for both 2013 and 2014. The final model is summarised in Table S3.2. These show the results are robust regardless of the cut-off points used.

**Table S3.2**: Summary of the output from the ordinal logistic regression model which include all nine covariates listed

| **2013** (3843 study subjects included in the analysis) | | | | **2014** (4040 study subjects included in the analysis) | | | |
| --- | --- | --- | --- | --- | --- | --- | --- |
| Covariates: | Wald statistic | P-value | Adjusted odds ratio  (95% CI) | Covariates: | Wald statistic | P-value | Adjusted odds ratio  (95% CI) |
| **Prior-year IV days**  **% predicted FEV1**  Chronic *P. aeruginosa*  Female  Age in years  CF related diabetes  Pancreatic insufficient  Intermittent *P. aeruginosa*  BMI in kg/m^2^  Centre 23 (vs Centre 28)*  Centre 15 (vs Centre 28)*  Centre 2 (vs Centre 28)*  Centre 11 (vs Centre 28)*  Centre 1 (vs Centre 28)* | **735.2**  **240.7**  49.6  33.8  25.8  13.4  8.8  7.4  4.3  5.4  4.9  4.7  4.1  3.9 | **< 0.001**  **< 0.001**  < 0.001  < 0.001  < 0.001  < 0.001  0.003  0.007  0.039  0.020  0.026  0.031  0.043  0.049 | 1.05 (1.05 – 1.06)  0.97 (0.97 – 0.98)  1.79 (1.52 – 2.11)  1.49 (1.30 – 1.71)  0.98 (0.97 – 0.99)  1.32 (1.14 – 1.53)  1.39 (1.12 – 1.72)  1.36 (1.09 – 1.71)  0.98 (0.97 – 0.99)  2.92 (1.18 – 7.24)  2.74 (1.13 – 6.67)  2.94 (1.11 – 7.81)  0.28 (0.08 – 0.96)  2.68 (1.00 – 7.16) | **Prior-year IV days**  **% predicted FEV1**  Chronic *P. aeruginosa*  Female  Pancreatic insufficient  Intermittent *P. aeruginosa*  Age in years  BMI in kg/m^2^  CF related diabetes  Centre 6 (vs Centre 28)*  Centre 20 (vs Centre 28)*  Centre 9 (vs Centre 28)*  Centre 5 (vs Centre 28)* | **719.1**  **230.2**  80.8  58.6  22.2  16.1  12.4  11.8  3.6  5.3  4.7  4.5  4.0 | **< 0.001**  **< 0.001**  < 0.001  < 0.001  < 0.001  < 0.001  < 0.001  0.001  0.057  0.021  0.030  0.033  0.045 | 1.05 (1.05 – 1.06)  0.97 (0.97 – 0.98)  2.05 (1.75 – 2.40)  1.67 (1.46 – 1.90)  1.66 (1.34 – 2.04)  1.53 (1.24 – 1.88)  0.99 (0.98 – 0.99)  0.97 (0.95 – 0.99)  1.15 (1.00 – 1.33)  0.40 (0.18 – 0.87)  0.41 (0.17 – 0.91)  0.40 (0.18 – 0.93)  0.41 (0.47 – 0.98) |

* Unlike a logistic regression model, the ordinal regression model is unable to determine the overall effect of CF centres on IV use. Therefore, 27 centres were compared against centre 28. Only centres with IV use that are significantly different from Centre 28 are shown in table C2.

*Sensitivity analysis 3: using four categories of IV days in an ordinal regression model*

Using current year IV days (no IV, 1-17 days, 18-42 days, ≥43 days) as the dependent variable in an ordinal logistic regression model with the same co-variates as described in the ‘Methods’ also yielded similar results. The final model is summarised in Table S3.3. These show the results with IV days are robust regardless of the cut-off points used.

**Table S3.3:** Summary of the output from the ordinal logistic regression model which include all nine covariates listed

| **2013** (3843 study subjects included in the analysis) | | | | **2014** (4040 study subjects included in the analysis) | | | |
| --- | --- | --- | --- | --- | --- | --- | --- |
| Covariates: | Wald statistic | P-value | Adjusted odds ratio  (95% CI) | Covariates: | Wald statistic | P-value | Adjusted odds ratio  (95% CI) |
| **Prior-year IV days**  **% predicted FEV1**  Chronic *P. aeruginosa*  Female  Age in years  CF related diabetes  Pancreatic insufficient  Intermittent *P. aeruginosa*  BMI in kg/m^2^  Centre 23 (vs Centre 28)*  Centre 11 (vs Centre 28)* | **784.9**  **228.8**  51.2  27.6  21.7  14.3  7.7  6.5  4.4  4.7  4.5 | **< 0.001**  **< 0.001**  < 0.001  < 0.001  < 0.001  < 0.001  0.006  0.011  0.036  0.031  0.034 | 1.05 (1.05 – 1.06)  0.97 (0.97 – 0.98)  1.82 (1.54 – 2.14)  1.44 (1.26 – 1.65)  0.98 (0.98 – 0.99)  1.34 (1.15 – 1.56)  1.36 (1.10 – 1.69)  1.34 (1.07 – 1.68)  0.98 (0.98 – 0.99)  2.73 (1.10 – 6.80)  0.26 (0.08 – 0.90) | **Prior-year IV days**  **% predicted FEV1**  Chronic *P. aeruginosa*  Female  Pancreatic insufficient  Intermittent *P. aeruginosa*  BMI in kg/m^2^  Age in years  CF related diabetes | **780.5**  **224.9**  70.3  40.1  23.2  13.8  9.5  6.7  2.9 | **< 0.001**  **< 0.001**  < 0.001  < 0.001  < 0.001  < 0.001  0.002  0.009  0.087 | 1.05 (1.05 – 1.06)  0.97 (0.97 – 0.98)  1.96 (1.68 – 2.30)  1.53 (1.34 – 1.75)  1.68 (1.36 – 2.08)  1.49 (1.21 – 1.83)  0.97 (0.95 – 0.99)  0.99 (0.98 – 1.00)  1.14 (0.98 – 1.32) |

* Unlike a logistic regression model, the ordinal regression model is unable to determine the overall effect of CF centres on IV use. Therefore, 27 centres were compared against centre 28. Only centres with IV use that are significantly different from Centre 28 are shown in table C3. In 2014, none of other adult CF centres differed significantly from Centre 28 in terms of IV days.

**Appendix S4: Sensitivity analyses using number of IV courses (instead of IV days) and different cut-offs to generate the clinical subgroups**

**Figure S4.1**: Tree-based diagram for 2013 to summarise current year IV days according to the different clinical subgroups using two categories of prior-year IV courses (≤ 1 course vs ≥ 2 courses)

**Adults with CF in 2013**

*n = 4269*

Median IV days in 2013 = 14 (IQR 0 – 35 days)

Jonckheere-Terpstra p-value for these three subgroups <0.001

Jonckheere-Terpstra p-value for these three subgroups <0.001

Mann-Whitney p-value for these two groups <0.001

**FEV_1_ < 40%**

*n = 490*

Median IV days in 2013

= 62

(IQR

36 – 98 days)

**FEV_1_ 40-69.9%**

*n = 634*

Median IV days in 2013

= 40

(IQR

18 – 58 days)

**FEV_1_ ≥ 70%**

*n = 280*

Median IV days in 2013

= 28

(IQR

14 – 42 days)

**FEV_1_ < 40%**

*n = 263*

Median IV days in 2013

= 14

(IQR

0 – 30 days)

**FEV_1_ 40-69.9%**

*n = 856*

Median IV days in 2013

= 5

(IQR

0 – 15 days)

**FEV_1_ ≥ 70%**

*n = 1385*

Median IV days in 2013

= 0

(IQR

0 – 9 days)

**Prior-year (i.e. 2012) ≥ 2 IV courses**

*n = 1477*

Median IV days in 2013 = 42 (IQR 23 – 70 days)

**Prior-year (i.e. 2012) ≤ 1 IV course**

*n = 2594*

Median IV days in 2013 = 0 (IQR 0 – 14 days)

**Figure S4.2**: Tree-based diagram for 2013 to summarise current year IV days according to the different clinical subgroups using four categories of prior-year IV courses (no IV, 1 course, 2-3 courses, ≥ 4 courses)

**Adults with CF in 2013**

*n = 4264*

Median IV days in 2013 = 14 (IQR 0 – 35 days)

Jonckheere-Terpstra

p-value for these four groups <0.001

**Prior-year (i.e. 2012) ≥4 IV courses** *n = 618*

Median IV days in 2013 = 67 (IQR 42 – 98 days)

**Prior-year (i.e. 2012) 2-3 IV courses** *n = 859*

Median IV days in 2013 = 28 (IQR 14 – 48 days)

**Prior-year (i.e. 2012) 1 IV course** *n = 806*

Median IV days in 2013 = 14 (IQR 0 – 27 days)

**Prior-year (i.e. 2012) no IV**

*n = 1788*

Median IV days in 2013 = 0

(IQR 0 – 8 days)

**FEV_1_**

**< 40%**

*n = 275*

Median IV days in 2013

= 80

(IQR

56 – 118 days)

Jonckheere-Terpstra p-value for these three subgroups <0.001

**FEV_1_**

**≥ 70%**

*n = 64*

Median IV days in 2013

= 34

(IQR

14 – 61 days)

**FEV_1_ 40-69.9%**

*n = 238*

Median IV days in 2013

= 56

(IQR

40 – 83 days)

**FEV_1_ 40-69.9%**

*n = 396*

Median IV days in 2013

= 28

(IQR

14 – 43 days)

Jonckheere-Terpstra p-value for these three subgroups <0.001

**FEV_1_**

**≥ 70%**

*n = 216*

Median IV days in 2013

= 28

(IQR

12 – 40 days)

**FEV_1_**

**< 40%**

*n = 215*

Median IV days in 2013

= 42

(IQR

25 – 63 days)

**FEV_1_ 40-69.9%**

*n = 339*

Median IV days in 2013

= 14

(IQR

0 – 28 days)

Jonckheere-Terpstra p-value for these three subgroups <0.001

**FEV_1_**

**≥ 70%**

*n = 324*

Median IV days in 2013

= 11

(IQR

0 – 17 days)

**FEV_1_**

**< 40%**

*n = 120*

Median IV days in 2013

= 15

(IQR

10 – 45 days)

Jonckheere-Terpstra p-value for these three subgroups <0.001

**FEV_1_ 40-69.9%**

*n = 517*

Median IV days in 2013

= 0

(IQR

0 – 14 days)

**FEV_1_**

**< 40%**

*n = 143*

Median IV days in 2013

= 4

(IQR

0 – 27 days)

**FEV_1_**

**≥ 70%**

*n = 1061*

Median IV days in 2013

= 0

(IQR

0 – 0 days)

**Figure S4.3**: Tree-based diagram for 2013 to summarise current year IV days according to the different clinical subgroups using four categories of prior-year IV days (no IV, 1-17 days, 18-42 days, ≥ 43 days)

**Adults with CF in 2013**

*n = 4264*

Median IV days in 2013 = 14 (IQR 0 – 35 days)

Jonckheere-Terpstra

p-value for these four groups <0.001

**Prior-year (i.e. 2012) ≥43 IV days** *n = 743*

Median IV days in 2013 = 60 (IQR 38 – 93 days)

**Prior-year (i.e. 2012) 18-42 IV days** *n = 791*

Median IV days in 2013 = 28 (IQR 14 – 44 days)

**Prior-year (i.e. 2012) 1-17 IV days** *n = 749*

Median IV days in 2013 = 14 (IQR 0 – 24 days)

**Prior-year (i.e. 2012) no IV**

*n = 1788*

Median IV days in 2013 = 0

(IQR 0 – 8 days)

**FEV_1_**

**< 40%**

*n = 317*

Median IV days in 2013

= 77

(IQR

51 – 115 days)

Jonckheere-Terpstra p-value for these three subgroups <0.001

**FEV_1_**

**≥ 70%**

*n = 82*

Median IV days in 2013

= 30

(IQR

14 – 56 days)

**FEV_1_ 40-69.9%**

*n = 298*

Median IV days in 2013

= 55

(IQR

35 – 79 days)

**FEV_1_ 40-69.9%**

*n = 369*

Median IV days in 2013

= 28

(IQR

14 – 42 days)

Jonckheere-Terpstra p-value for these three subgroups <0.001

**FEV_1_**

**≥ 70%**

*n = 204*

Median IV days in 2013

= 28

(IQR

13 – 41 days)

**FEV_1_**

**< 40%**

*n = 190*

Median IV days in 2013

= 42

(IQR

21 – 60 days)

**FEV_1_ 40-69.9%**

*n = 306*

Median IV days in 2013

= 14

(IQR

0 – 28 days)

Jonckheere-Terpstra p-value for these three subgroups <0.001

**FEV_1_**

**≥ 70%**

*n = 318*

Median IV days in 2013

= 11

(IQR

0 – 16 days)

**FEV_1_**

**< 40%**

*n = 103*

Median IV days in 2013

= 14

(IQR

10 – 35 days)

Jonckheere-Terpstra p-value for these three subgroups <0.001

**FEV_1_ 40-69.9%**

*n = 517*

Median IV days in 2013

= 0

(IQR

0 – 14 days)

**FEV_1_**

**< 40%**

*n = 143*

Median IV days in 2013

= 4

(IQR

0 – 27 days)

**FEV_1_**

**≥ 70%**

*n = 1061*

Median IV days in 2013

= 0

(IQR

0 – 0 days)

**Figure S4.4**: Tree-based diagram for 2014 to summarise current year IV days according to the different clinical subgroups using two categories of prior-year IV courses (≤ 1 course vs ≥ 2 courses)

**Adults with CF in 2014**

*n = 4644*

Median IV days in 2014 = 14 (IQR 0 – 34 days)

Jonckheere-Terpstra p-value for these three subgroups <0.001

Jonckheere-Terpstra p-value for these three subgroups <0.001

Mann-Whitney p-value for these two groups <0.001

**FEV_1_ < 40%**

*n = 494*

Median IV days in 2014

= 56

(IQR

30 – 91 days)

**FEV_1_ 40-69.9%**

*n = 630*

Median IV days in 2014

= 40

(IQR

20 – 60 days)

**FEV_1_ ≥ 70%**

*n = 298*

Median IV days in 2014

= 28

(IQR

14 – 43 days)

**FEV_1_ < 40%**

*n = 270*

Median IV days in 2014

= 14

(IQR

0 – 37 days)

**FEV_1_ 40-69.9%**

*n = 911*

Median IV days in 2014

= 8

(IQR

0 – 17 days)

**FEV_1_ ≥ 70%**

*n = 1485*

Median IV days in 2014

= 0

(IQR

0 – 9 days)

**Prior-year (i.e. 2013) ≥ 2 IV courses**

*n = 1501*

Median IV days in 2014 = 42 (IQR 21 – 69 days)

**Prior-year (i.e. 2013) ≤ 1 IV course**

*n = 2760*

Median IV days in 2014 = 0 (IQR 0 – 14 days)

**Figure S4.5**: Tree-based diagram for 2014 to summarise current year IV days according to the different clinical subgroups using four categories of prior-year IV courses (no IV, 1 course, 2-3 courses, ≥ 4 courses)

**Adults with CF in 2014**

*n = 4644*

Median IV days in 2014 = 14 (IQR 0 – 34 days)

Jonckheere-Terpstra

p-value for these four groups <0.001

**Prior-year (i.e. 2013) ≥4 IV courses** *n = 588*

Median IV days in 2014 = 66 (IQR 42 – 98 days)

**Prior-year (i.e. 2013) 2-3 IV courses** *n = 913*

Median IV days in 2014 = 28 (IQR 14 – 49 days)

**Prior-year (i.e. 2013) 1 IV course** *n = 902*

Median IV days in 2014 = 14 (IQR 0 – 28 days)

**Prior-year (i.e. 2013) no IV**

*n = 1858*

Median IV days in 2014 = 0

(IQR 0 – 8 days)

**FEV_1_**

**< 40%**

*n = 263*

Median IV days in 2014

= 73

(IQR

51 – 112 days)

Jonckheere-Terpstra p-value for these three subgroups <0.001

**FEV_1_**

**≥ 70%**

*n = 59*

Median IV days in 2014

= 43

(IQR

28 – 58 days)

**FEV_1_ 40-69.9%**

*n = 217*

Median IV days in 2014

= 56

(IQR

37 – 75 days)

**FEV_1_ 40-69.9%**

*n = 413*

Median IV days in 2014

= 28

(IQR

14 – 49 days)

Jonckheere-Terpstra p-value for these three subgroups <0.001

**FEV_1_**

**≥ 70%**

*n = 239*

Median IV days in 2014

= 21

(IQR

11 – 41 days)

**FEV_1_**

**< 40%**

*n = 231*

Median IV days in 2014

= 37

(IQR

20 – 61 days)

**FEV_1_ 40-69.9%**

*n = 387*

Median IV days in 2014

= 14

(IQR

0 – 28 days)

Jonckheere-Terpstra p-value for these three subgroups <0.001

**FEV_1_**

**≥ 70%**

*n = 349*

Median IV days in 2014

= 10

(IQR

0 – 15 days)

**FEV_1_**

**< 40%**

*n = 137*

Median IV days in 2014

= 26

(IQR

14 – 42 days)

Jonckheere-Terpstra p-value for these three subgroups <0.001

**FEV_1_ 40-69.9%**

*n = 524*

Median IV days in 2014

= 0

(IQR

0 – 14 days)

**FEV_1_**

**< 40%**

*n = 133*

Median IV days in 2014

= 11

(IQR

0 – 28 days)

**FEV_1_**

**≥ 70%**

*n = 1136*

Median IV days in 2014

= 0

(IQR

0 – 0 days)

**Figure S4.6**: Tree-based diagram for 2014 to summarise current year IV days according to the different clinical subgroups using four categories of prior-year IV days (no IV, 1-17 days, 18-42 days, ≥ 43 days)

**Adults with CF in 2014**

*n = 4644*

Median IV days in 2014 = 14 (IQR 0 – 34 days)

Jonckheere-Terpstra

p-value for these four groups <0.001

**Prior-year (i.e. 2013) ≥43 IV days** *n = 770*

Median IV days in 2014 = 60 (IQR 40 – 92 days)

**Prior-year (i.e. 2013) 18-42 IV days** *n = 791*

Median IV days in 2014 = 28 (IQR 14 – 42 days)

**Prior-year (i.e. 2013) 1-17 IV days** *n = 842*

Median IV days in 2014 = 14 (IQR 0 – 28 days)

**Prior-year (i.e. 2013) no IV**

*n = 1858*

Median IV days in 2014 = 0

(IQR 0 – 8 days)

**FEV_1_**

**< 40%**

*n = 325*

Median IV days in 2014

= 70

(IQR

47 – 107 days)

Jonckheere-Terpstra p-value for these three subgroups <0.001

**FEV_1_**

**≥ 70%**

*n = 85*

Median IV days in 2014

= 43

(IQR

28 – 61 days)

**FEV_1_ 40-69.9%**

*n = 302*

Median IV days in 2014

= 52

(IQR

29 – 72 days)

**FEV_1_ 40-69.9%**

*n = 359*

Median IV days in 2014

= 28

(IQR

14 – 42 days)

Jonckheere-Terpstra p-value for these three subgroups <0.001

**FEV_1_**

**≥ 70%**

*n = 230*

Median IV days in 2014

= 16

(IQR

8 – 35 days)

**FEV_1_**

**< 40%**

*n = 182*

Median IV days in 2014

= 33

(IQR

21 – 56 days)

**FEV_1_ 40-69.9%**

*n = 356*

Median IV days in 2014

= 14

(IQR

0 – 28 days)

Jonckheere-Terpstra p-value for these three subgroups <0.001

**FEV_1_**

**≥ 70%**

*n = 332*

Median IV days in 2014

= 10

(IQR

0 – 15 days)

**FEV_1_**

**< 40%**

*n = 124*

Median IV days in 2014

= 24

(IQR

9 – 42 days)

Jonckheere-Terpstra p-value for these three subgroups <0.001

**FEV_1_ 40-69.9%**

*n = 524*

Median IV days in 2014

= 0

(IQR

0 – 14 days)

**FEV_1_**

**< 40%**

*n = 133*

Median IV days in 2014

= 11

(IQR

0 – 28 days)

**FEV_1_**

**≥ 70%**

*n = 1136*

Median IV days in 2014

= 0

(IQR

0 – 0 days)
